# Hepatocyte-Specific Knock-Out of Nfib Aggravates Hepatocellular Tumorigenesis via Enhancing Urea Cycle

**DOI:** 10.3389/fmolb.2022.875324

**Published:** 2022-05-17

**Authors:** Li Zhou, Qing-Liang Wang, Lin-Hong Mao, Si-Yuan Chen, Zi-Han Yang, Xue Liu, Yu-Hua Gao, Xiao-Qin Li, Zhi-Hang Zhou, Song He

**Affiliations:** ^1^ Department of Gastroenterology, The Second Affiliated Hospital of Chongqing Medical University, Chongqing, China; ^2^ Department of Pathology, The Second Affiliated Hospital of Chongqing Medical University, Chongqing, China; ^3^ Department of Gastroenterology, Chengdu Second People’s Hospital, Sichuan, China; ^4^ Department of Biomedical Science, City University of Hong Kong, Hong Kong, China; ^5^ Department of Pathology, College of Basic Medicine, Jining Medical University, Jining, China; ^6^ Key Laboratory of Precision Oncology in Universities of Shandong, Institute of Precision Medicine, Jining Medical University, Jining, China

**Keywords:** nuclear factor I/B, hepatocellular carcinogenesis, urea cycle, conditional knockout, CRISPR/Cas9

## Abstract

Nuclear Factor I B (NFIB) has been reported to promote tumor growth, metastasis, and liver regeneration, but its mechanism in liver cancer is not fully elucidated. The present study aims to reveal the role of NFIB in hepatocellular carcinogenesis. In our study, we constructed hepatocyte-specific NFIB gene knockout mice with CRISPR/Cas9 technology (Nfib^−/−^; Alb-cre), and induced liver cancer mouse model by intraperitoneal injection of DEN/CCl_4_. First, we found that Nfib^−/−^ mice developed more tumor nodules and had heavier livers than wild-type mice. H&E staining indicated that the liver histological severity of Nfib^−/−^ group was more serious than that of WT group. Then we found that the differentially expressed genes in the tumor tissue between Nfib^−/−^ mice and wild type mice were enriched in urea cycle. Furthermore, ASS1 and CPS1, the core enzymes of the urea cycle, were significantly upregulated in Nfib^−/−^ tumors. Subsequently, we validated that the expression of ASS1 and CPS1 increased after knockdown of NFIB by lentivirus in normal hepatocytes and also promoted cell proliferation *in vitro*. In addition, ChIP assay confirmed that NFIB can bind with promoter region of both ASS1 and CPS1 gene. Our study reveals for the first time that hepatocyte-specific knock-out of Nfib aggravates hepatocellular tumor development by enhancing the urea cycle.

## Graphical Abstract

Schematic diagram of NFIB promoting chemo-induced hepatocellular carcinoma by affecting urea cycle rate-limiting enzymes ASS1 and CPS1. Specific knockout of liver NFIB leads to increased expression of ASS1 and CPS1, which promotes the occurrence of liver cancer by affecting the urea cycle.
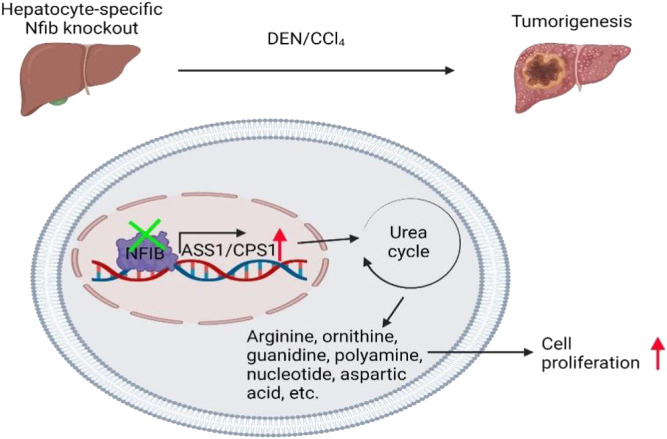

## Introduction

Nuclear factor I B (NFIB) is one of the four NFI family members. At present, most of the research on it focuses on the growth and development of various tissues and organs. NFIB was highly expressed in the embryonic lung and mice with NFIB null mutant die early postnatally and exhibit severe lung hypoplasia (Grunder et al., 2002). Also, NFIB-null mice were defective in development of brain, including the basilar pons and hippocampus (Wang et al., 2007; Mason et al., 2009). NFIB haploinsufficiency caused intellectual disability and macrocephaly (Schanze et al., 2018). Hair follicle stem-cell-specific conditional targeting of NFIB in mice uncoupled stem cell synchrony by promoting melanocyte stem cell proliferation and differentiation (Chang et al., 2013). More recently, conditional knock out of NFIB in skin stem cells led to the loss of their hair-regenerating capability (Adam et al., 2020). On the other side, loss of NFIB expression resulted in prostatic hyperplasia (Grabowska et al., 2016). Besides, NFIB could inhibit the HBV (Guo et al., 2011) and HIV replication (Vemula et al., 2015). Roy and colleagues found that NFIB could promote liver cell regeneration (Roy et al., 2017). In the context of tumor, the role of NFIB is tissue-specific. On the one hand, NFIB promoted the proliferation and inhibited apoptosis of triple-negative breast cancer (Liu R.-Z. et al., 2019; Zilli et al., 2021), myeloproliferative tumors (Rice et al., 2011), colorectal cancer (Liu Z. et al., 2019), and renal cancer (Wang et al., 2021). On the other hand, it could inhibit the malignant phenotypes of osteosarcoma (Mirabello et al., 2015), cutaneous squamous cell carcinoma (Zhou et al., 2015) and lung adenocarcinoma (Becker-Santos et al., 2016). More recently, Chen and colleagues demonstrated that mice with loss of function NFIB mutant alleles developed more gliomas and have shortened survival time (Chen et al., 2021).

Hepatocellular carcinoma (HCC), the fifth most common cancer world-wide and the second cause of tumor-associated death, progresses through a series of steps, including the reprogramming of cellular metabolism (Marquardt and Thorgeirsson, 2014). The development of HCC is associated with virus infection, alcohol, liver steatosis, and aflatoxin. As mentioned above, NFIB can inhibit HBV replication and promote liver regeneration. Also, Zhang and colleagues reported that NFIB promotes HCC growth (Zhang et al., 2015). It is suggested that NFIB can not only enhance the self-healing of hepatocytes, but also promote the growth of variant tumors. Therefore the mechanism of its transformation from a protective factor to a cancer promoting factor in the liver needs to be clarified.

Hepatocytes process toxic ammonia produced by protein and amino acid metabolism. The urea cycle (UC) converts intracellular ammonia into urea, which is then excreted into the urine. The UC process mainly happens in liver. There were five UC catalytic enzymes involved in hepatic nitrogen disposal: CPS1, OTC, ASS1, ASL and ARG (Keshet et al., 2018). In contrast to the role of UC enzymes in liver nitrogen disposal, mounting evidence suggests that UC intermediates are shunted to anabolic routes in cancer. Multiple types of tumors exhibit altered expression levels of UC components. CPS1, the first-rate limiting enzyme, is upregulated in a subset of non-small cell lung cancer cells and support the biosynthesis of pyrimidines and promote cancer cell proliferation (Celiktas et al., 2017). However, low expression of CPS1 was associated with short overall survival time of HCC patients (Ridder et al., 2021). ASS1, another key enzyme in UC, not only promoted the growth and invasion of gastric cancer (Tsai et al., 2018) and colorectal cancer (Doubleday et al., 2021), but also inhibited the progression of HCC (Kim et al., 2021). Additionally, some of the products of UC, such as NO and pyrimidine, has been reported to promote tumor initiation (Lala and Chakraborty, 2001; Evageliou et al., 2016; Novita Sari et al., 2021). Therefore, the mechanism of ASS1 and CPS1 in liver cancer is still unclear, and further studies are needed.

In the present study, we successfully generated NFIB hepatocyte-specific knock-out mice and found that knockout of Nfib dramatically augmented hepatocellular tumorigenesis in DEN/CCL_4_-induced model. Moreover, gene expression profiling showed that urea cycle enzymes were significantly upregulated in Nfib-knockout tumors. Subsequent validation confirmed that the limiting enzymes CPS1 and ASS1 were upregulated in Nfib-knockout tumors. Moreover, knockdown of NFIB in the hepatocyte cell line L02 significantly increased the expression of CPS1 and ASS1. Taken together, the present study demonstrated that hepatocellular NFIB mitigates hepatocellular tumorigenesis via depressing urea cycle.

## Materials and Methods

### Animals and Conditional Knock Out of Nuclear Factor I B

Mice with homozygous conditional-null alleles of NFIB (Nfib^f/f^) were generated with the help of the Cyagen Biosciences (Guangzhou, China). Briefly, guide RNA (gRNA), Cas9 mRNA and the donor vector targeting the exon 1 site was microinjected into mouse zygotes to create a NFIB-flox allele (Nfib^f/f^) with two LoxP sites fanking exon 1. Nfib^f/f^ mice were bred to Alb-Cre mice (Jackson Laboratory, Bar Harbor, ME;003574) to generate hepatocyte-specifc NFIB-knockout mice (Nfib^f/f^ Alb-Cre). Wild-type (WT) mice were purchased from Beijing Vital River Laboratory. Primers for genotyping are listed in Supplementary Table S1. All mice were C57BL/6 background. Under the standard laboratory conditions of Chongqing Medical University (Chongqing, China), all mice had free access to food and water and lived in a 12-h light-dark cycle environment. All experiments preformed were approved by the Research Ethics Committee of Chongqing Medical University and conducted according to the institutional guidelines (No. 2021-489).

### Chemically Inducing Liver Tumors

For chemically induced HCC, sex-matched mice (*n* = 16 per group) aged 14–16 days were given a single intraperitoneal (i.p.) injection of DEN (Sigma, 75 mg/kg) and two subsequent biweekly injections of CCl_4_ (Sigma, 2 ml/kg, 1:4 diluted with olive oil, i.p., starting 1 week after DEN and lasted for 12 weeks), The sample size was referred to relevant literature, and the individuals which died in the middle of the study were not included in the analysis. Mice were sacrificed at the indicated time points and specific samples were taken for further analysis. The mice were sacrificed and fresh livers were separated, washed in ice PBS for three times to remove the blood clots and connective tissue on the surface, and the livers were weighed and the number of tumors visible to the naked eye was counted. The tissue was then immobilized in 4% paraformaldehyde for more than 24 h, embedded in paraffin and sectioned continuously.

### Histology and Immunohistochemistry

Paraffin sections were obtained from the Department of Pathology, Jiangnan District, the Second Affiliated Hospital of Chongqing Medical University. To determine tumor burden and pathology, sections of each liver was stained with haematoxylin and eosin (H&E), mounted with NFIB, ASS1, CPS1 and PCNA using a chromogenic immunohistochemistry method. Primary antibodies and dilutions used were listed in Supplementary Table S2. Sections were deparaffinized in xylene, followed by rehydration in an ethanol gradient. For HE staining, the nucleus was first stained with hematoxylin, then differentiated with 1% hydrochloric acid alcohol for a few seconds, followed by eosin cytoplasm staining, and then dehydrated and sealed. For immunohistochemistry (IHC), Endogenous peroxidase was blocked with 3% hydrogen peroxide in distilled water, and sodium citrate antigenic repair solution (BOSTER, AR0024) was microwaved-heated for 10 min for three times. Then washing with PBS buffer (BOSTER, AR0031) for three times. Afterwards, sections were incubated overnight at 4°C with primary antibodies against NFIB (1:300, #ab186738, Abcam), ASS1(1:200, #16210-1-AP, proteintech), CPS1(1ug/ml, #ab45956, Abcam). After three repeated washings with PBS sections were incubated with Biotin-labeled secondary antibody solution (UltraSensitive™ SP IHC Kit, Maxim) for 10 min and covered with *streptomyces* antibiotic protein-peroxidase reagent (UltraSensitive™ SP IHC Kit, Maxim) for 10 min at room temperature. Sections were stained in DAB kit (Color development kit, ZLI-9018, ZSGB-BIO) and counterstained with hematoxylin. Finally, sections were covered with neutral balsam (G8590, Solarbio). PCNA (1:200, proteintech, #Cat 10205-2-AP) was stained as previously described. Image acquisition was performed at a magnification of 20× with CaseViewer2. 4. Z1 microscope, Axiocam MRm and HRc cameras using Axiovision 4.8 software (Carl Zeiss, Inc., Oberkochen, Germany).

### RNA-Seq and Bioinformatic Analysis

The transcriptome sequencing and analysis were conducted by OE biotech Co., Ltd. (Shanghai, China). Total RNA was extracted using the mirVana miRNA Isolation Kit (Ambion) following the manufacturer’s protocol. RNA integrity was evaluated using the Agilent 2100 Bioanalyzer (Agilent Technologies, Santa Clara, CA, United States). The samples with RNA Integrity Number (RIN) ≥7 were subjected to the subsequent analysis. The libraries were constructed using TruSeq Stranded mRNA LTSample Prep Kit (Illumina, San Diego, CA, United States) according to the manufacturer’s instructions. Then these libraries were sequenced on the Illumina sequencing platform (HiSeqTM 2500 or Illumina HiSeq × Ten) and 125 bp/150 bp paired-end reads were generated. Raw data (raw reads) were processed using Trimmomatic. The reads containing ploy-N and the low-quality reads were removed to obtain the clean reads. Then the clean reads were mapped to reference genome using hisat2. FPKM value of each gene was calculated using cufflinks, and the read counts of each gene were obtained by htseq-count. DEGs were identified using the DESeq (2012) R package functions estimateSizeFactors and nbinomTest. *p* value < 0.05 and fold change >2 or foldChange <0.5 was set as the threshold for significantly differential expression. Hierarchical cluster analysis of DEGs was performed to explore genes expression pattern. GO enrichment and KEGG pathway enrichment analysis of DEGs were respectively performed using R based on the hypergeometric distribution.

### Agarose Gel Electrophoresis

Total RNA was extracted from mouse tail (mouse tail direct PCR kit, B40013, Bimake, United States) and used as template for amplification to obtain DNA samples. Goldview nucleic acid dye (DH392-5, Dingguo, China) was added for AGAR gel electrophoresis. Finally, the gel was exposed using an exposure instrument (Champchemi TM580).

### Cell Culture

The human HCC cell lines Huh7, HepG2, SMMC-7721, SK-Hep1, PLC/PRF/5 and normal hepatocyte L02 were obtained from the American Type Culture Collection. SMMC-7721 and L02 were obtained from National biomedical experimental cell resource bank (BMCR). All cells in the experiment were maintained in Dulbecco’s Modified Eagle Medium (HyClone, Logan, UT, United States). All cell lines used in this study were tested for the absence of *Mycoplasma* contamination every 2 weeks. All culture media were supplemented with 10% FBS (Gibco, Rockville, MD, United States), 100 units/mL penicillin, and 100 μg/ml streptomycin (HyClone) in a humidifed incubator of 5% CO2 and 95% air at 37°C.

### Lentivirus Infected Cells and Establishment of Stable Transgenic Strains

L02 and SK-Hep1 cells grown in logarithmic phase were inoculated into 6-well plates and cultured in 5% CO2 incubator at 37°C. After adding puromycin with different concentration gradients, the cell death was observed, and the lowest drug concentration leading to all cell death was selected as the working concentration of puromycin. Then, the logarithmic growth cells were inoculated into a 6-well plate and placed in a cell incubator cultivation. Lentivirus infection (Heyuan Biotechnology, Shanghai) was carried out when the cell growth density reached 70%–80%. After 24 h, the medium was replaced with fresh medium containing 10% serum. 72 hours after the cells were infected with lentivirus, the fluorescence of the cells was observed under the fluorescence microscope, accounting for more than 90% of the cell volume. According to the selected working concentration of puromycin, an appropriate amount of puromycin was added to the culture medium, and a new puromycin containing culture medium was changed every 2 days. Puromycin continuously screened cells for 1 week, extracted cell RNA, detected cell infection efficiency by qRT-PCR, and established a stable transfected cell line.

### RNA Extraction and Quantitative Real-Time PCR

Total RNA was isolated from cell lines with RNAiso Plus (Takara, cat#9109, Japan). For qRT-PCR, complementary DNA (cDNA) was synthesized using a HiFiScript gDNA Removal RT MasterMix (CWBIO) according to the manufacturer’s protocol with primers for genes defined in RNA-seq. qRT-PCR analysis was performed using 2 × SYBR Green Master Mix (Thermo Fisher Scientific) on CFX96TM Real-time PCR System (Bio-Rad Laboratories). Samples were subjected to 95°C for 30 min, 45 cycles of amplification at 95°C for 5 s and 60°C for 30 s, melt curve 65°C–95°C increment 0.5°C for 5 s. We do each reaction three times. Target gene expression levels were standardized by using the glyceraldehyde-3-phosphate dehydrogenase (GAPDH) gene as an internal reference. The mRNA levels of each target gene were normalized to the mRNA levels of GAPDH and expressed as 2^ΔCt^ (ΔCt = Ct target–Ct GAPDH). The relative quantity of the mRNA levels of the tested genes was determined by the following equation: Relative quantity = 1,000/2^ΔCt^. The primers for qRT-PCR are listed in Supplementary Table S1.

### Immunofluorescence Staining

Cells in a confocal dish were treated for 48 h, fixed with 4% paraformaldehyde, treated with Triton X-100, sealed with goat serum, incubated with primary antibodies (anti-NFIB, ASS1, CPS1) and secondary antibodies, stained with DAPI, and observed with a confocal laser scanning microscope (Leica, Germany). Main antibody information is shown in Supplementary Table S2.

### Protein Extraction and Western Blot Analysis

The protein was extracted by Radioimmunoprecipitation assay (RIPA) buffer. The supernatant of cell lysates was run on a 10% acrylamide gel by SDS-PAGE and then transferred to a polyvinylidene fluoride membrane (Millipore). Anti-NFIB (1:1,000, #ab186738, Abcam), Anti-ASS1 (1:1,000, #16210-1-AP, proteintech), Anti-CPS1 (1:1,000, #ab45956, Abcam) and Anti-GAPDH (1:1,000, #ab8245, Abcam) were combined with HRP-conjugated secondary antibody (1:1,000) for western blotting. A chemiluminescence western blotting detection system (Bio-Rad) was used for protein detection.

### Chromatin Immunoprecipitation Assay

Cells were harvested and cross-linked with 37% paraformaldehyde for 10 min. After incubation with 0.125 M glycine to terminate the cross-linking, the cells were lysed using a lysis buffer (1 M Dithiothreitol, 200× Protease Inhibitor Cocktail), followed by incubation for 10 min on ice. After centrifugation at 2000×g, the nuclei of the cells were digested in 0.5 μl Micrococcal Nuclease and sonicated to shear the DNA to a size range of 150–900 bp. Then, 30 μl Protein G Magnetic Beads (Cell Signaling Technology, United States) were added to the mixture. After washes with cold high/low salt lotion, the mixture was incubated with anti-NFIB antibody (1:30, #ab186738, Abcam) or control immune-globulin G (IgG) overnight at 4°C. Subsequently, the beads were eluted with elution buffer (100 mM NaHCO3 and 1% SDS). The eluate was incubated with 150 μl 1× ChIP at 65°C for 30 min and then with a buffer (6 μl 5 M NaCl, proteinase K, pH 8.0) at 65°C for 2 h, followed by DNA purification with a centrifugal column at 18,500 × g for 30 s.

### Statistical Analysis

All measurement data were verified to conform to normal distribution by Shapiro-Wilk test. All values are expressed as mean ± standard deviation (SD). All data were obtained from at least three repetitions of each experiment. Statistical significance was analyzed between the treatment and control groups using GraphPad Prism 8.0 software (La Jolla, CA, United States). Statistical significance was determined by Student’s t-test was used to compare two groups except sequencing data. Probability values (*p*) less than 0.05 was considered statistically significant.

## Results

### Construction of Hepatocyte-Specific Nuclear Factor I B Knockout Mice

**FIGURE 1:**
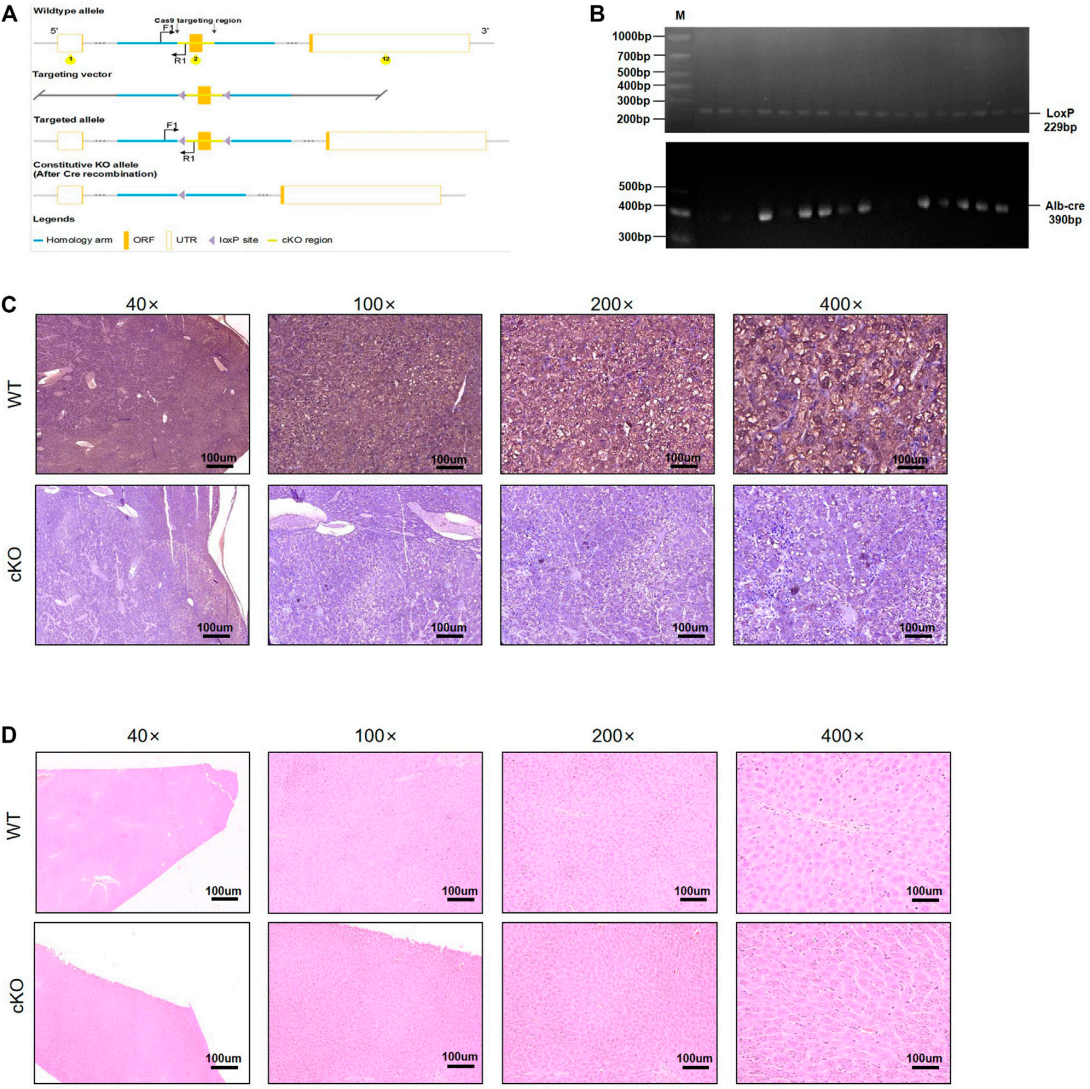
Generation of hepatocyte-specific NFIB-knockout mice (Nfib^f/f^ Alb-Cre). **(A)** NFIB gene knockout targeting vector strategy design diagram. **(B)** Genotyping of Nfib^loxp/loxp^ Alb-cre (Nfib^f/f^ Alb-Cre) mice using the tail DNA. The above panel shows the homozygous loxp mice. The wild-type (WT) allele yields an amplicon of 162 bp, while the floxed allele yields an amplicon of 229 bp. The panel below shows the Alb-Cre positive mice, with an amplicon of 390 bp. **(C)** IHC analysis of NFIB protein in the livers of WT and Nfib^f/f^ Alb-Cre mice. **(D)** H&E staining showing the histology of the livers of WT and Nfib^f/f^ Alb-Cre mice. All scale bars = 100 μm. Original magnification×20. IHC, immunohistochemistry.

The CRISPR/Cas9 system is emerging as a powerful tool for engineering the genome in diverse organisms. As an RNA-guided DNA endonuclease, Cas9 can be easily programmed to target new sites by altering its guide RNA sequence, making sequence-specific gene editing several magnitudes easier (Wang et al., 2016). The gRNA was designed to target the first exon, which is shared by all the NFIB variants (Supplementary Figure S1A). The strategy is shown in Supplementary Figure S1B. Conditional knockout mice were produced by injecting the NFIB gene knockout target vector and Cas9 mRNA into the eggs of fertilized C57BL/6 mice (Figure 1A). After confirming the correct insertion of loxP site, the conditional knockout (cKO) mice were hybridized with wild-type mice to produce offspring. PCR and sequencing identification results and cultivation plan are shown in Supplementary Figure S2A–C. After the successful construction of Nfib-floxed chimeric mice (Nfib^f/f^), the mice were hybridized with Alb-Cre mice to obtain hepatocyte-specific NFIB knockout mice (Nfib^f/f^ Alb-Cre). Genotyping was performed on the offspring mice (Figure 1B). Supplementary Table S1 listed all the primer information. The loss of NFIB expression in liver was further validated by immunohistochemical staining in liver section from Nfib^f/f^ Alb-Cre mice and wild-type mice (Figure 1C). H&E staining showed that the liver histology was not affected after knocking out NFIB (Figure 1D). Taken together, we successfully constructed hepatocyte-specific NFIB-knock-out (Nfib^f/f^ Alb-Cre) mice by using CRISPR/Cas9 technology.

### Hepatocyte-Specific Knock-Out of Nfib Promotes Hepatocellular Tumorigenesis

**FIGURE 2:**
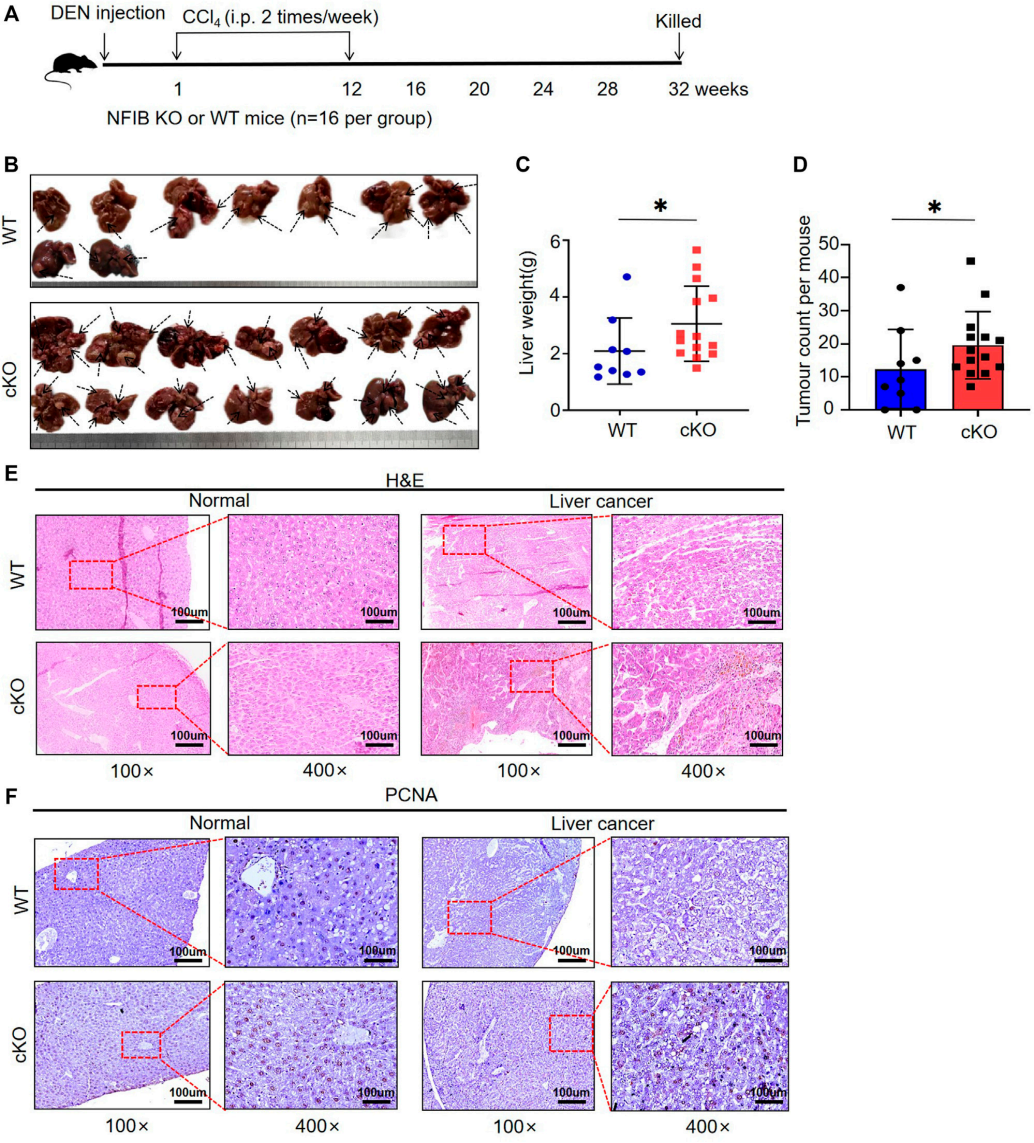
Hepatocyte-specific knock-out of NFIB promotes hepatocellular tumorigenesis. **(A)** Overview of experimental design. Cohorts of cKO (*n* = 16) and WT (*n* = 16) mice aged 14–16 days were administered DEN/CCl_4_. Liver samples were collected at 32 weeks after DEN exposure for histopathological analysis. **(B)** Gross appearance of livers from the indicated mice. Compared to WT, cKO mice display multiple and more diffuse tumor masses. As the arrow shows. The number of independent tumors was determined for each mouse. Each tumor mass was considered an independent tumor if it was separated from another tumor mass by normal tissue. **(C,D)** Number of tumors and liver weight. On average, cKO (*n* = 14) mice had more tumors and higher liver weight than WT (*n* = 9) (bar indicates mean; Mann-Whitney test: *, *p* < 0.05). **(E)** Histology of murine hepatocellular neoplasms. Representative photomicrographs of serial sections of normal liver tissue and liver tumors arising in DEN/CCl_4_-treated mice. H&E staining demonstrates tissue morphology. **(F)** The PCNA staining of the tissues. All scale bars = 100 μm. Original magnification × 20. DEN, diethylnitrosamine; H&E, haematoxylin and eosin; HCC, hepatocellular carcinoma. *, *p* < 0.05.

Next, we studied the role of hepatic NFIB in a DEN/CCl_4_ HCC model of Nfib^f/f^ Alb-Cre (cKO) and wild-type (WT) mice. Randomized sex-matched WT (*n* = 16) and cKO (*n* = 16) mice were injected with DEN (25 mg/kg, i.p.) at the age of 14–16 days followed by 24 injections of CCl_4_ (2 ml/kg, i.p., twice a week for 12 weeks, starting 1 week after DEN injection). Mice were sacrificed 8 months after DEN injection (Figure 2A). Seven and two mice died due to the toxicity of the drugs within the first 2 months in the WT and cKO group, respectively. Representative gross appearance of the liver with tumors is shown in Figure 2B. The number of tumor nodules observed by naked eye in cKO mice was significantly higher than that in WT mice (10.89 ± 2.82 vs. 19.57 ± 2.73, *p* = 0.046) (Figure 2C). The liver weight in cKO mice were also higher than those in WT mice (3.41 ± 2.10 vs.9.77 ± 2.42, *p* = 0.039) (Figure 2D). Histopathological analysis showed that liver tumors in cKO mice were more disordered and heteromorphic than those in WT mice, with unclear nuclei, a large number of vacuole-like degeneration structures, and more vigorous cell division and proliferation (Figure 2E). PCNA staining was stronger in NFIB cKO tumor tissue, indicating higher proliferating activity (Figure 2F). These results showed that loss of hepatic NFIB increases the tumorigenesis of DEN/CCl_4_-induced HCC.

### Urea Cycle Was Dramatically Enhanced in Nuclear Factor I B Knock-Out Tumors

**FIGURE 3:**
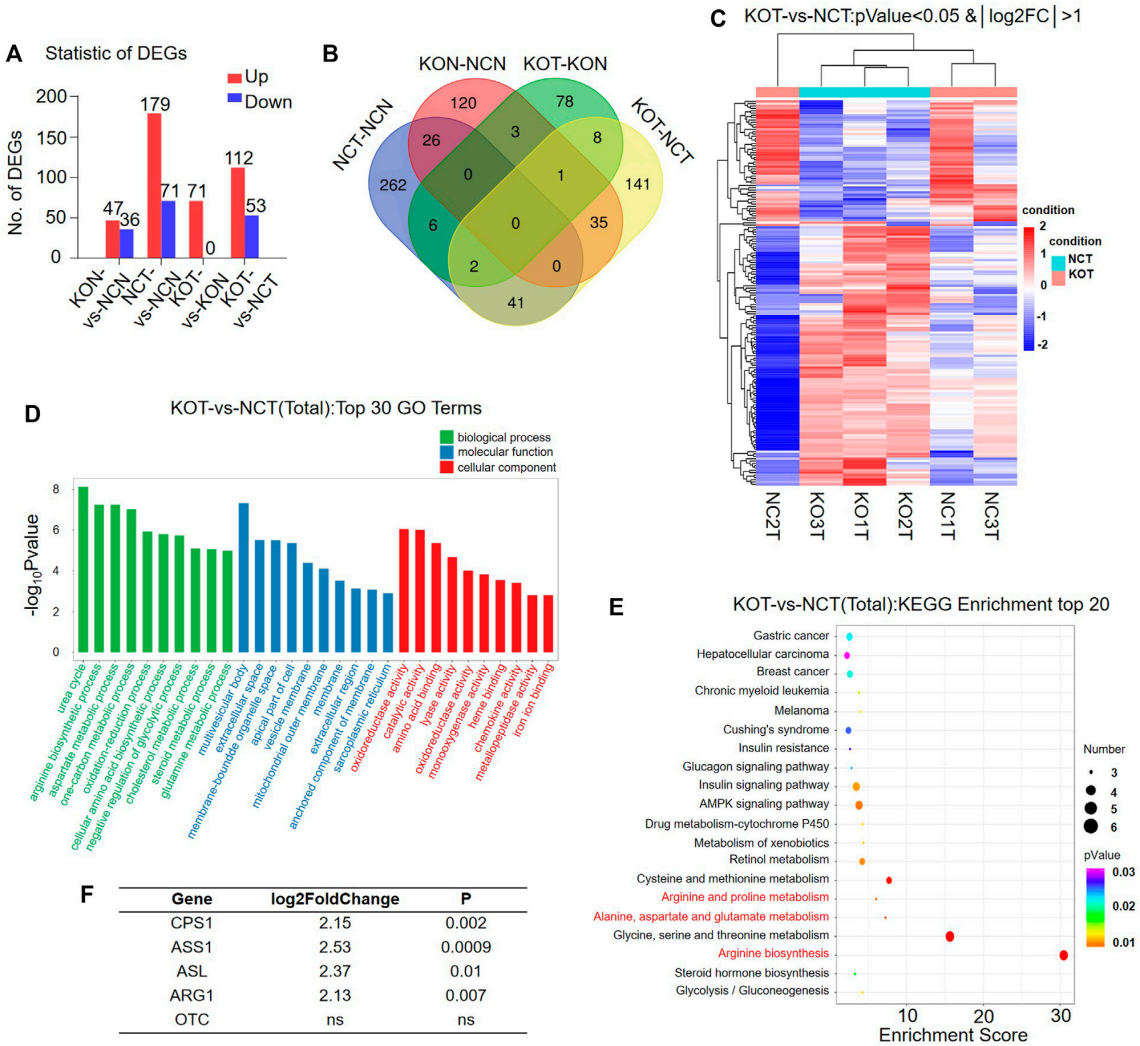
Urea cycle is remarkably activated in Nfib^−/−^ liver tumors. **(A)** The number of differentially expressed genes (DEGs) in mice in a pair-wise comparison. **(B)** Venn diagram showed the overlaping of DEGs in different groups. **(C)** Heatmap diagrams showed the relative expression levels of total DEGs among the KOT vs. NCT group. The upregulated genes are in read, and the downregulated genes are in green. **(D)** GO analysis of DEGs between KOT and NCT in three main categories. **(E)** KEGG pathway enrichment analysis of the annotated DEGs. **(F)** The foldchange of urea cycle enzymes between KOT and NCT tumors.

Urea cycle enzymes are involved in tumor metabolic reprogramming (Kim et al., 2017). The specific expression of a variety of UC enzymes in hepatocellular carcinoma (HCC) meets the needs of tumor cells for different UC intermediates (Su et al., 2018). We divided the livers of Nfib^f/f^ Alb-Cre (*n* = 3, male) and WT (*n* = 3, male) mice into four groups: KOT (cKO Tumor), KON (cKO Normal), NCT (WT Tumor), NCN (WT Normal). Pairwise comparison was performed (KOT-vs- NCT, KON-vs-NCN, KOT-vs-KON, NCT-vs-NCN) for mRNA sequencing analysis. Differentially expressed genes (DEGs) were then analyzed. A total of 4,174 DEGs were identified, of which 83, 250, 71 and 165 DEGs were found between KON and NCN, NCT and NCN, KOT and KON, and KOT and NCT, respectively (Figure 3A). Venn diagram was used to show the overlap between groups (Figure 3B). A general overview of the DEGs between KOT and NCT group was shown in a heatmap (Figure 3C). The detailed information of the top 10 DEGs were shown in Table 1 and the DEGs were then evaluated with GO (Figure 3D) and KEGG pathway analyses (Figure 3E). In the biological process category, the DEGs were significantly enriched in “Urea cycle”, “aspartate metabolic process” and “arginine biosynthetic process”. In the cellular component category, “multivesicular body” was significantly enriched. In the molecular function category, “oxidoreductase activity”, “catalytic activity” and “amino acid binding” were significantly enriched. In the KEGG analysis, these DEGs were mostly enriched in “Alanine, aspartate and glutamate metabolism”, “Arginine biosynthesis”, “AMPK signaling pathway”, “Arginine and proline metabolism”, “Hepatocellular carcinoma” and “Gastric cancer”. Alanine, aspartate, glutamate, and arginine are all intermediate products of UC cycle. Four of the five urea cycle enzymes, CPS1, ASS1, ASL and ARG1, were significantly upregulated in KOT group (Figure 3F). The enrichment results of DEGs between tumor and para-tumor tissues were shown in Supplementary Figure S3A–D. Interestingly, we found that the expression of urea cycle enzymes (CPS1, ASS1, ASL) were also increased in Nfib-knockout livers (Table 2). These results showed that NFIB knockout promotes urea cycle in the tumor tissue by up-regulating CPS1 and ASS1 expression.

**TABLE 1:** Top10 of DEGs between KOT and NCT group.

Upregulated			Downregulated		
Gene ID	log2FoldChange	*p*-value	Gene ID	log2FoldChange	*p*-value
Myh8	11.11783086	0.028925511	Muc3	−8.179469725	0.008232239
Cyp11a1	6.8781355	0.033892884	Cxcl17	−4.964470331	0.006566219
Jsrp1	5.906525142	0.048501534	Muc4	−4.774725753	2.03539E-05
Myadml2	5.338306042	0.020850979	Npc1l1	−4.601098972	0.0216155
Klhl38	5.244663002	0.012700445	Spire2	−4.571306249	0.025320444
Elane	5.111884477	0.039555279	Tmed6	−4.448123323	0.034421725
Art1	4.987316718	0.04859742	Cst8	−4.345725557	0.022300874
Baiap2l2	3.897111174	0.04181349	Slc24a3	−4.205899015	0.027910738
Obscn	3.844088105	0.000826451	Mep1a	−3.844765665	0.012123581
Col25a1	3.624649771	0.016011977	Omp	−3.6000286	0.022541822

**TABLE 2:** The lipid metabolism and urea cycle related genes altered in NFIB knock out liver.

Gene	Biological process	Fold change	*p*
ACSS2	Lipid biosynthetic process	1.85	0.028
FASN	Fatty acid synthesis	1.73	0.000
PNPLA3	Lipid catabolic process	1.71	0.035
ADORA1	Lipid catabolic process	1.61	0.029
CES1G	Lipid catabolic process	1.59	0.028
Mid1ip1	Lipid biosynthetic process	1.54	0.010
ACLY	Lipid biosynthetic process	1.53	0.006
CES3a	Lipid catabolic process	0.57	0.038
Insig2	Inhibiting cholesterol biosynthesis	0.56	0.001
ACS2	Lipid catabolic process	0.32	0.027
CES4a	Lipid catabolic process	0.06	0.005
CPS1	Urea cycle	1.69	0.007
ASS1	Urea cycle	1.51	0.007
ASL	Urea cycle	1.63	0.019
ARG1	Urea cycle	ns	ns
OTC	Urea cycle	ns	ns

### Lipid Metabolism Was Changed in Nuclear Factor I B Knock-Out Liver

**FIGURE 4:**
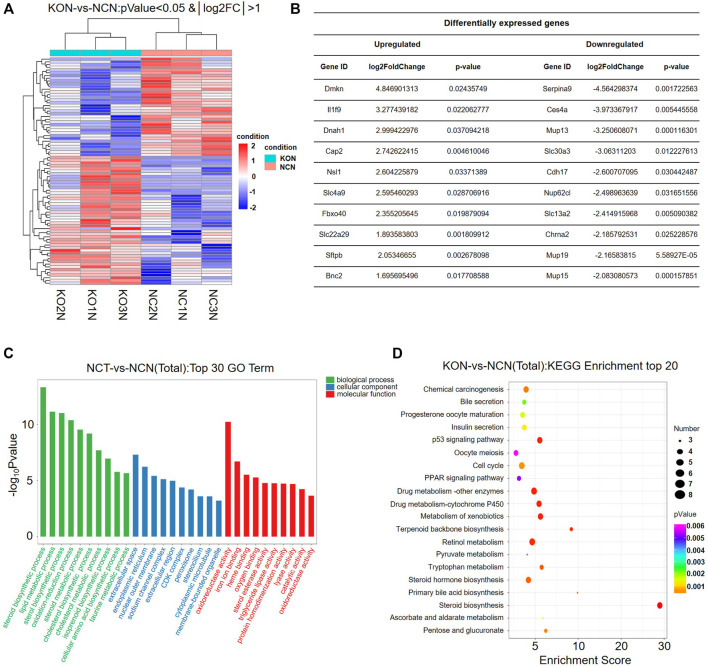
Lipid metabolism pathway is significantly altered in Nfib^−/−^ liver. **(A)** Heatmap diagrams showed the relative expression levels of total DEGs among the KON vs. NCN group, deep red = upregulated expression; white = no difference in expression; deep blue = downregulated expression. **(B)** Table of DEGs (upregulated and downregulated) in the top 10. **(C)** GO analysis of DEGs between KON and NCN in three main categories. The DEGs are dramatically enriched in lipid metabolism pathway. **(D)** KEGG pathway enrichment analysis of the annotated DEGs.

To reveal the effect of NFIB knock out in the non-cancerous tissue, we further analyze the DEGs between KON and NCN groups (Figure 4A). Detailed DEGs are shown in Figure 4B. GO enrichment analysis revealed that the DEGs are significantly enriched in biological process including lipid metabolism, steroid synthesis, sterol synthesis and oxidative-reduction (Figure 4C). KEGG enrichment showed that those DEGs are significantly enriched in chemical carcinogenesis, bile secretion, insulin secretion, p53 pathway, cell cycle and PPAP signaling pathway (Figure 4D). The DEGs that are involved in lipid metabolism include ACSS2, FASN, PNPLA3, ADORA1, CES1G, Mid1ip1, ACLY, CES3a, Insig2, ACS2, CES4a (Table 2). Interestingly, 3 of the 5 urea cycle enzymes were also upregulated in the NFIB cKO liver, although to a lesser degree compared with that in tumor tissues (Table 2). These results implied that NFIB may regulate lipid metabolism and urea cycle in liver.

### Nuclear Factor IB Decreased the Expression of CPS1 and ASS1 in HCC Tissues and Hepatocytes, and Inhibited the Proliferation of Hepatocytes

**FIGURE 5:**
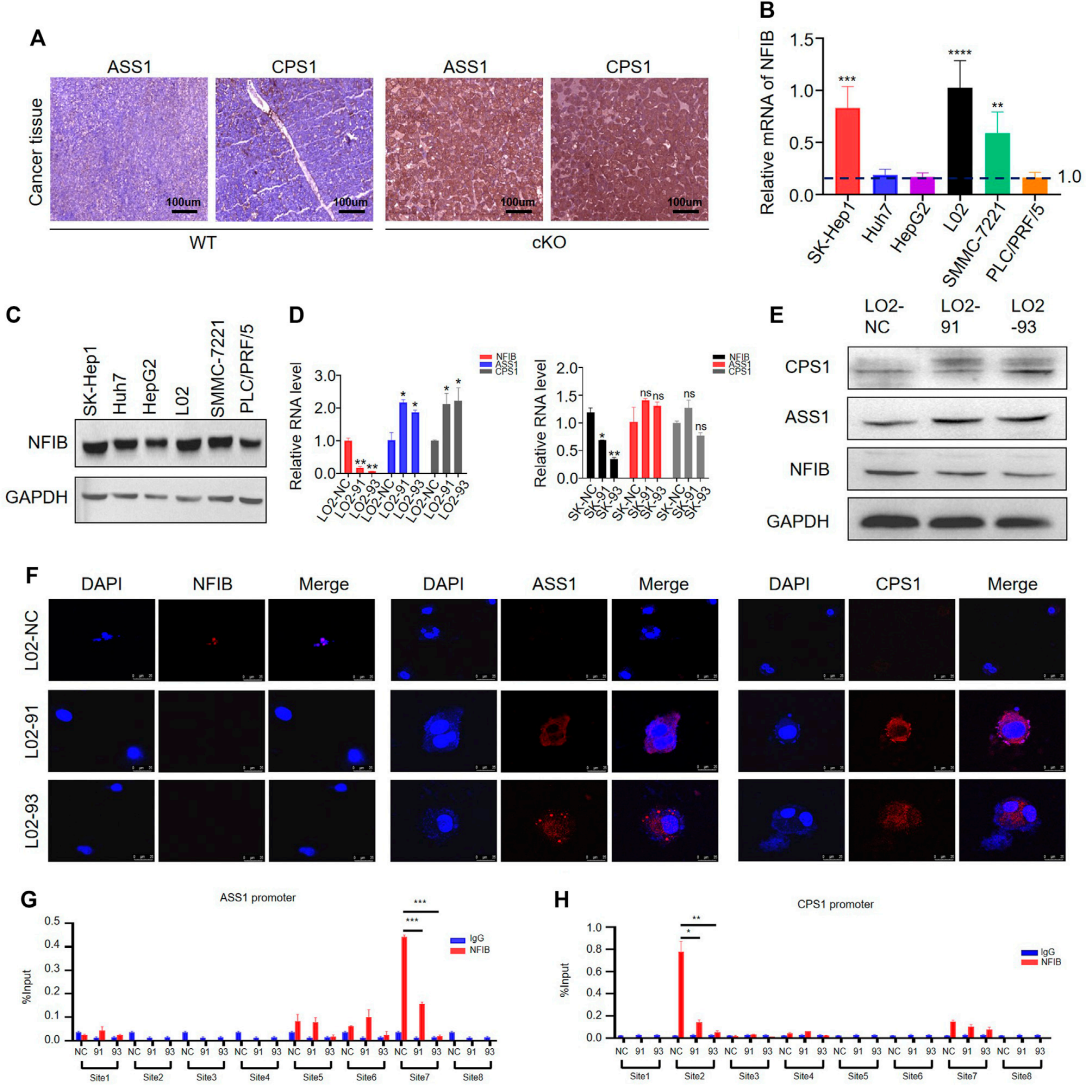
NFIB inhibits CPS1 and ASS1 expression in hepatocytes. **(A)** IHC staining shows that both CPS1 and ASS1 are upregulated in Nfib^−/−^ cancer liver. **(B)** The expression level of NFIB in HCC cell lines and L02 by RT-PCR. **(C)** The expression level of NFIB in HCC cell lines and L02 by Western blot. **(D)** Knocking down NFIB significantly increased the expression of ASS1 and CPS1 expression in normal hepatocyte L02 by qPCR. **(E)** Knocking down NFIB significantly increased the expression of ASS1 and CPS1 expression in normal hepatocyte L02 but not in SK-Hep1 cells by western blot. The NFIB knocking down cells with two different shRNAs were respectively designated as 91 and 93. The corresponding scramble one was assigned with NC. **(F)** Immunofluorescence staining showing the increased expression of ASS1 and CPS1. **(G,H)** ChIP assay for the ASS1 and CPS1 promoters. L02 cells were transduced with NFIB-knockdown lentivirus or control, and ChIP was performed by IP with either anti-NFIB antibody (red) or control IgG (blue). Real-time PCR analysis of relative binding affinity of NFIB to the eight binding sites in ASS1 promoter region. ***, *p* < 0.001. Real-time PCR analysis of relative binding affinity of NFIB to the eight binding sites in CPS1 promoter region. All scale bars = 100 μm. Original magnification × 20. *, *p* < 0.05; **, *p* < 0.01.

Based on the sequencing analyses, we found that multiple rate-limiting enzymes associated with the urea cycle (CPS1, ASS1, ASL) were upregulated in Nfib^f/f^ Alb-Cre livers and liver tumor tissues (Table 2). In order to verify this result, we performed immunohistochemical detection on the liver cancer tissues of mice. The expressions of ASS1 and CPS1 in the livers were increased after NFIB knockout compared with the control group (Figure 5A), which was consistent with the sequencing results. We then wondered whether knockdown of NFIB would cause the same results in human liver cancer or normal liver cell lines. First, we verified the expression of NFIB in a variety of HCC cell lines and normal liver cells (Figures 5B,C). qPCR and WB results showed that NFIB expression was higher in SK-Hep1 and L02 cells. Therefore, we transfected SK-Hep1 HCC cell and normal hepatocyte L02 with lentivirus containing shRNA sequence to stably knock down NFIB. The protein level of ASS1 and CPS1 were significantly increased in NFIB-knockdown L02 cells but not in SK-Hep1 cells (Supplementary Figures S4A, Figure 5D,E). Immunofluorescence staining also confirmed this result (Figure 5F). Further, CCK8 assay was performed on L02 with control or knockdown sequence. L02 cell proliferation increased significantly after knocking down NFIB (Supplementary Figure S4B).

As a member of the NFI family of transcription factors, NFIB can directly regulate gene transcription (Wang et al., 2021). In order to verify whether NFIB or NFI family has binding sites with ASS1 and CPS1, we first search the NCBI (http://jaspar.genereg.net/), UCSC (UCSC Genome Browser Home http://genome.ucsc.edu/) database and Promoter of 2.0 Prediction of software (http://www.cbs.dtu.dk/services/Promoter/) to find ASS1, CPS1 promoter regions, joint Jaspar database (http://jaspar.genereg.net/) to forecast the transcription factor binding sites (Supplementary Figure S4C). It was found that there are NFI binding sites in the promoter region of both ASS1 and CPS1 (Supplementary Figure S4D). We then performed the chromatin immunoprecipitation (ChIP) assay to test whether NFIB could bind with the promoter region of ASS1 and CPS1. Cell lysates were sonicated to generate ∼150 bp chromatin fragments prior to immunoprecipitation. Primer Premier6.0 software was used to design 8 pairs of ASS1 and CPS1 primers for real-time PCR amplification of ChIP products (Supplementary Table S3). ASS1 promoter region have three proximal (−504 to −624 bp, −620 to −749 bp and −723 to −878 bp) and one distal (−1,696 to −1800 bp) putative NFIB binding sites (Figure 5G). CPS1 promoter region has one proximal (−542 to −661 bp) and three distal (−1,049 to −1,167 bp, −1,049 to −1,193 bp and −1,210 to −1,390 bp) putative NFIB binding sites (Figure 5H). The schematic diagram of the primers designed by ASS1 and CPS1 in the promoter region is shown in Supplementary Figure S5A,B. In combination with the results from NFIB-knockdown cells, we found that the immunoprecipitation of only the ASS1 distal site (site 7) and CPS1 proximal (site 2) was remarkably decreased in NFIB-knockdown cells. Overall, existing results showed that NFIB directly binds to the ASS1 and CPS1 promoters in liver cells. Taken together, these results suggested that NFIB binds with the promoter region and decreases the expression of ASS1 and CPS1 to inhibit hepatocyte proliferation.

## Discussion

NFIB plays an important role in regulating various developmental and physiological processes, and there is growing evidence that NFIB is associated with a range of malignancies (Becker-Santos et al., 2017). In 2016, Denny et al. found that NFIB plays a carcinogenic role in small cell lung cancer (SCLC) and is a key molecule involved in driving the metastasis of SCLC (Denny et al., 2016). Most recently NFIB has been shown to mediate a highly invasive and migratory phenotype in melanoma, where it directly promotes EZH2 expression, also leading to changes in the chromatin state of tumor cells to facilitate this aggressive behavior (Fane et al., 2017). But little is known about the role of NFIB in carcinogenesis. Only one study reported the effect of NFIB in carcinogenesis, where NFIB deficiency exacerbated the development of high-grade glioma (Chen et al., 2021). Herein, we generated hepatocyte-specific NFIB knock-out mice and found that NFIB inhibits hepatocellular tumorigenesis in a DEN/CCl_4_ model. It seems that the effect of NFIB in tumor biology is stage-specific. Consistently, it has been reported that tyrosine phosphatase Shp2 not only enhances liver cancer progression but also suppresses hepatocellular carcinogenesis (Han et al., 2015; Liu et al., 2018). In addition, we also found that NFIB can promote the growth and survival of HCC cells (data not shown).

Further transcriptomic sequencing revealed that NFIB knock out leads to upregulation of urea cycle enzymes both in tumor tissue and normal liver tissue. Urea cycle is critical for hepatocytes to detoxify by eliminating intracellular ammonia. These enzymes also participate in tumor progression. ASS1 produces arginine succinic acid by breaking down aspartate. On the one hand, ASS1 can promote cancer progression. ASS1 is elevated in lung, colon, gastric and ovarian cancers, and supports cell proliferation and metastasis (Delage et al., 2010). On the other hand, ASS1 can inhibit tumor progression. ASS1 deficiency can redirect aspartate to foster pyrimidine synthesis and promote the proliferation of tumor cells (Rabinovich et al., 2015). Reduction in ASS1 expression has been postulated as an independent prognostic biomarker in pancreatic cancer and sarcomas (Zheng et al., 2013). Latest study revealed that ASS1 can prevent HCC progression by activating the ATF4/CHOP axis (Kim et al., 2021). The expression of ASS1 can be inhibited by HIF1α in lung cancer cells (Long et al., 2016) and by promoter methylation (Syed et al., 2013). The role of CPS1 in tumor progression is also double-faced. CPS1 has been reported to promote maintain pyrimidine pools and DNA synthesis and thus facilitate tumor growth in lung cancer (Cardona et al., 2009). The tumor suppressor P53 can directly inhibit the transcription of CPS1 and promoter hypermethylation is proposed to reduce its expression (Liu et al., 2011). However, CPS1 is downregulated in HCC and associated with poor prognosis (Ridder et al., 2021). In addition, other studies have found that the metabolism of fatty acid oxidation (FAO) in liver cancer cells is active, and the activation of transcription factor FOXM1 can promote tumorigenesis. After the use of fatty acid inhibitor etomoxir (ETO), it can significantly inhibit the growth of CPS1 deficient tumor cells, and improve the sensitivity of tumor cells to sorafenib (Wu et al., 2021). Although accumulating evidence has revealed their role in tumor progression, their role in tumor initiation remains unknown. We found that knockdown of NFIB can only augment the expression of CPS1 and ASS1 in normal hepatocyte but not in hepatoma cell, implying its stage-specific effect. Therefore, we suspected that hepatoma cell lines may be different from normal hepatocytes in metabolic mechanism. During the transformation of hepatocytes into hepatoma cell, the regulatory effect of NFIB on ASS1 and CPS1 changed. Moreover, urea cycle enzymes (ASS1 and CPS1) are inhibited in liver cancer (Kim et al., 2021; Ridder et al., 2021), so the expression of them have decreased in liver cancer cells, and the increase of ASS1 and CPS1 caused by knocking down NFIB becomes insignificant. We propose that ASS1 and CPS1 might be necessary for hepatocellular tumorigenesis and early-stage cancer to detoxify, but reduce in the advanced stage when tumor cells are faced with hostile microenvironment.

NFIB can directly or indirectly regulate gene transcription. Previous studies have shown that NFIB can directly promote the transcription of EZH2 (Piper et al., 2014), IGFBP5 (Perez-Casellas et al., 2009), or inhibit the transcription of p21 in TP53-mutated triple-negative breast cancer (Liu R.-Z. et al., 2019), CDK6 and CDK4 (Zhou et al., 2015). It can also regulate gene expression by regulating chromatin accessibility (Denny et al., 2016). Moreover, NFIB could increase HER2 expression *via* upregulating circMAP7D1 in gastric cancer cells (Yang et al., 2021). In this study we found that the promoter regions of ASS1 and CPS1 had binding sites for the NFIB, probably further inhibiting their transcription.

Here we provide evidence showing that NFIB, a nuclear gene expression regulator, links urea cycle to the liver cancer. Hepatocyte-specific knock-out of Nfib aggravates hepatocellular tumorigenesis *via* upregulating key urea cycle enzymes such as ASS1 and CPS1. It is expected to provide a new strategy for the treatment of liver cancer by analyzing the effect of NFIB on the metabolic changes and tumorigenesis mechanism of liver cancer.

## Data Availability

The datasets presented in this study can be found in online repositories. The names of the repository/repositories and accession number(s) can be found in the article. Supplementary Material.
