# Supplementary material for: Hepatocyte-Specific Knock-Out of Nfib Aggravates Hepatocellular Tumorigenesis via Enhancing Urea Cycle
Source: Front Mol Biosci. 2022 May 17;9:875324. doi: 10.3389/fmolb.2022.875324 (PMC9152321; doi:10.3389/fmolb.2022.875324)
Supplement: Supplementary file 1 [file Table1.PDF]

Supplementary Tables

Table S1. Primer sequences for qPCR, genotyping and sequencing confirmation thestudy.

Gene name	Forward 5'-3'	Reverse 5'-3'
**qPCR**		
Human GAPDH	5'-CCTTCCGTGTCCCCACT-3'	5'-GCCTGCTTCACCACCTTC-3'
Human NFIB	5'-CTGGAAGTCGAACATGGC-3'	5'-GGGAAGAATCCTGTGGAGA-3'
Human ASS1	5'-TCGTGCATCCTCGTGTG-3'	5'-CCTTCTTCCTGGCTTCCTC-3'
Human CPS1	5'-AGAAATGGACGCTGTTGG-3'	5'-CCTTGGCTGATGGTTTGT-3'
**Genotyping**		
NFIB	5'-TGTTTCAGTGTTGGAATGTTGGACG-3'	5'-GGTGGCACAGAAACACAAAGCATG-3'
Alb-Cre	5'-GAAGCAGAAGCTTAGGAAGATGG-3'	5-TTGGCCCCTTACCATAACTG-3'
**Sequencing Confirmation**		
Loxp 5'Sequence primer (F3)	5'-GATGAATGCATGCTGGAAGCTAATG-3'	
Loxp 3'Sequence primer (F4)	5'-TCTGACAGAGGCCTAGATTTATGTTG-3'	

Table S2. The primary antibodies and dilutions used in the study.

**Gene name**	**Forward 5'-3'**	**Reverse 5'-3'**
**qPCR**		
Human GAPDH	5'-CCTTCCGTGTCCCCACT-3'	5'-GCCTGCTTCACCACCTTC-3'
Human NFIB	5'-CTGGAAGTCGAACATGGC-3'	5'-GGGAAGAATCCTGTGGAGA-3'
Human ASS1	5'-TCGTGCATCCTCGTGTG-3'	5'-CCTTCTTCCTGGCTTCCTC-3'
Human CPS1	5'-AGAAATGGACGCTGTTGG-3'	5'-CCTTGGCTGATGGTTTGT-3'
**Genotyping**		
NFIB	5'-TGTTTCAGTGTTGGAATGTTGGACG-3'	5'-GGTGGCACAGAAACACAAAGCATG-3'
Alb-Cre	5'-GAAGCAGAAGCTTAGGAAGATGG-3'	5-TTGGCCCCTTACCATAACTG-3'
**Sequencing Confirmation**		
Loxp 5'Sequence primer (F3)	5'-GATGAATGCATGCTGGAAGCTAATG-3'	
Loxp 3'Sequence primer (F4)	5'-TCTGACAGAGGCCTAGATTTATGTTG-3'	

Table S3. ChIP primer sequences of ASS1 and CPS1.

Gene name	Sense	AntiSense
**ASS1**		
Site1	CTCATCCTTACTCGGCTACCA	GCTTCGTGCTCACTTCTATCC
Site2	TTCTCATCCTTACTCGGCTACC	GCTTCGTGCTCACTTCTATCC
Site3	TCCTGTCTAGTGGGTTCGC	CTGATTGCTGGCCTGTCTT
Site4	CCTCCTGTCTAGTGGGTTCG	CTGATTGCTGGCCTGTCTT
Site5	CATACAGGGTAGTCGTGAAGGT	CAGGAGGCAAGTGAGGAACA
Site6	TCCGCTCTGCCACTCACAT	TTTCACCTTCACGACTACCCTG
Site7	GCGGCAAGGAGTAAAGGTC	CAGCATGTGAGTGGCAGAG
Site8	AGTCCTCCCTCACCTTGGT	CTCCTTGCCGCAGAAATGG
**CPS1**		
Site1	GTCAGCCTCACTCTCTCCTAT	TTTGGGATGCTAGCCTCAG
Site2	GCAGAAGTGAGGACCTAGAATC	CGAACAGCAGAAATACCAGCAT
Site3	GGACAGTAACAGAAGGGCTATT	ACCCTGCGTTATCCTTTAGAAA
Site4	GGACAGTAACAGAAGGGCTATT	ACCCTGCGTTATCCTCTAGAGC
Site5	GGACAGTAACAGAAGGGCTATT	ACGCCTGTGTCTTTAACAAACT
Site6	GGACAGTAACAGAAGGGCTATT	ACGGCGAAGGCTCTGATT
Site7	GGGATGCTCAACTCTCTCAG	CCGTAGGTTACATTGATGCT
Site8	CGTCCTTACCATACACCACC	GGTGAAGAATCCTAACGGTATT
