# Supplementary material for: Hepatocyte-Specific Knock-Out of Nfib Aggravates Hepatocellular Tumorigenesis via Enhancing Urea Cycle
Source: Front Mol Biosci. 2022 May 17;9:875324. doi: 10.3389/fmolb.2022.875324 (PMC9152321; doi:10.3389/fmolb.2022.875324)
Supplement: Supplementary file 2 [file Image1.PDF]

Supplementary Figures

Figure S1. Schematic representation of the strategy to generate the NFIB conditional allele targeting the second exon region.

Figure S2. PCR and sequencing identification results and cultivation plan.

Figure S3. GO and KEGG pathway enrichment analysis of the annotated DEGs between KOT-vs-KON and NCT-vs-NCN.

Figure S4. L02 knockdown of NFIB affected ASS1 and CPS1 expression and inhibited cell proliferation. The NFI family has binding sites with ASS1 and CPS1.

Figure S5. Diagram representing the position of primers used for PCR in the ASS1 and CPS1 promoter regions.

A

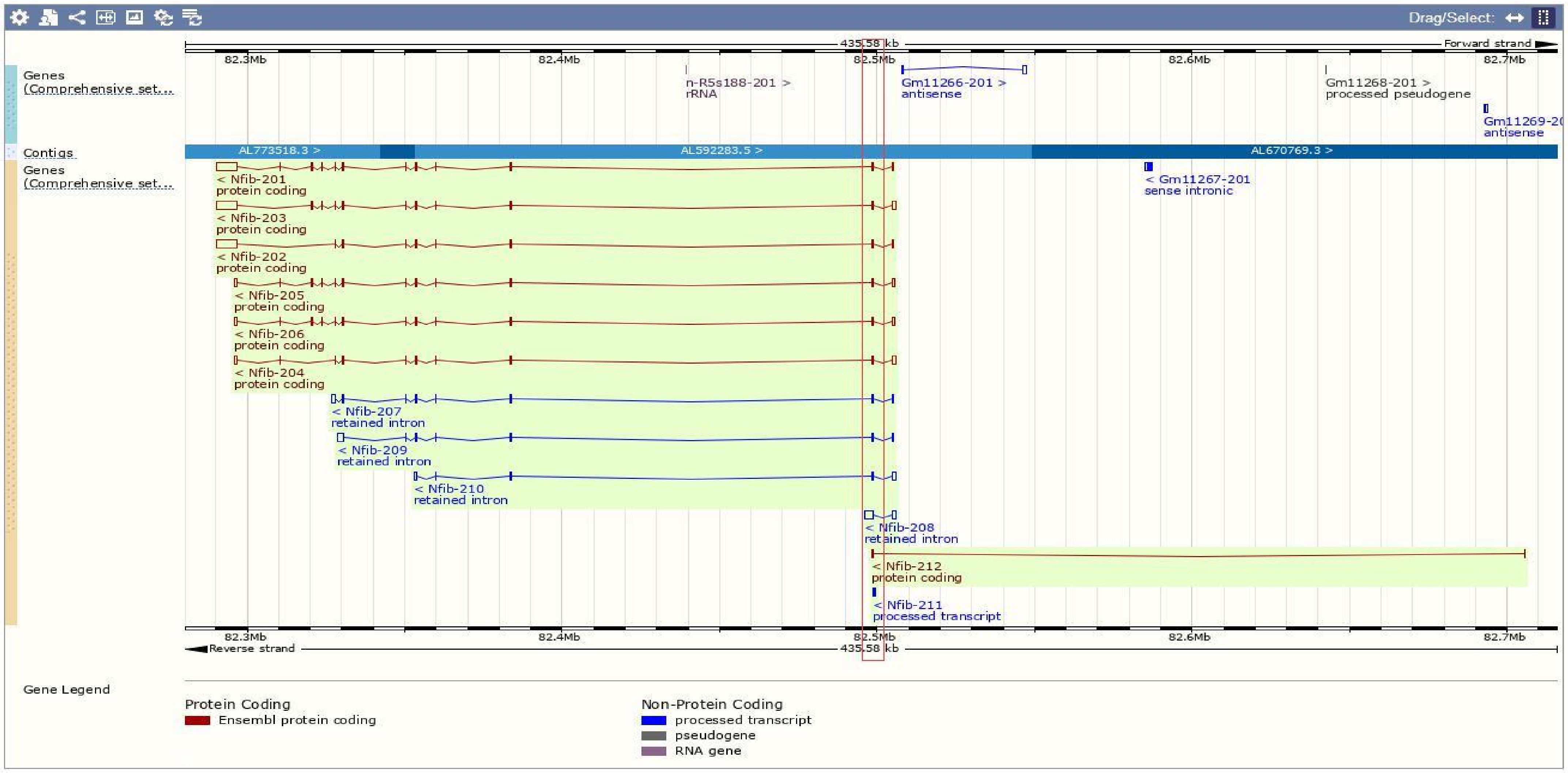

B

Schematic depiction of targeting strategy

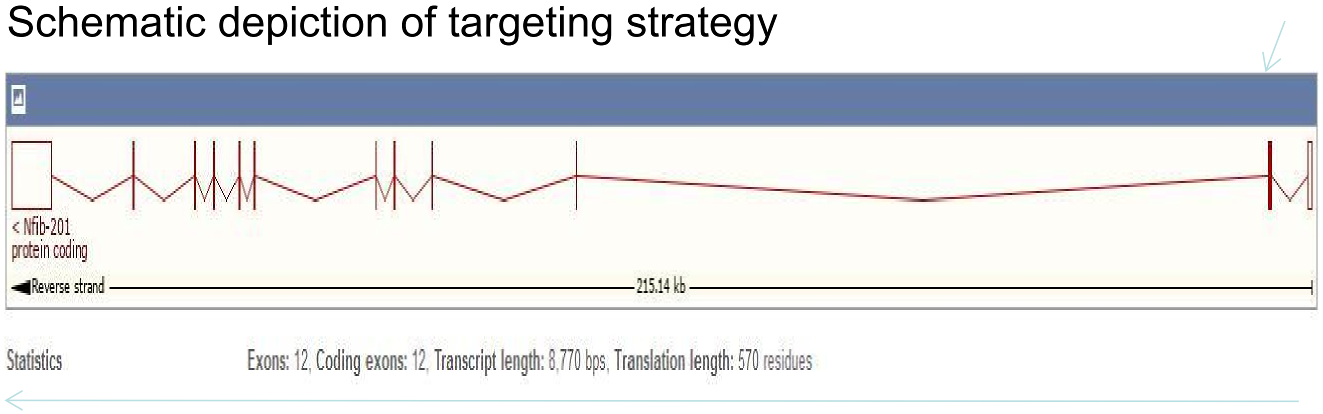

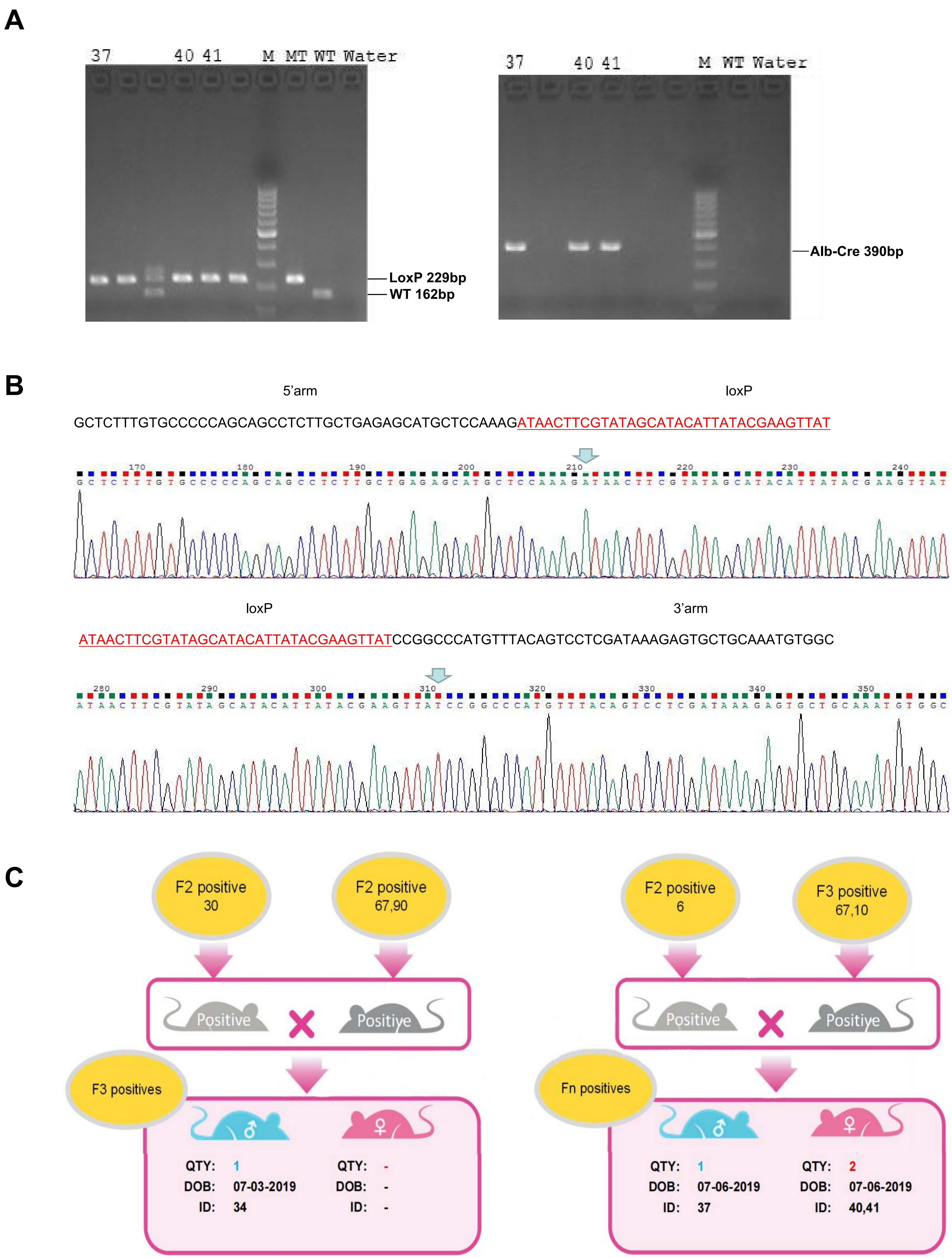

A
37
40 41
M MT WT Water
LoxP 229bp
WT 162bp
37
40 41
M WT Water
Alb-Cre 390bp
B
5'arm
loxP
GCTCTTTGTGCCCCCAGCAGCCTCTTGCTGAGAGCATGCTCCAAAGATAACTTCGTATAGCATACATTATACGAAGTTAT
loxP
3'arm
ATAACTTCGTATAGCATACATTATACGAAGTTATCCGGCCCATGTTTACAGTCCTCGATAAAGAGTGCTGCAAATGTGGC
C
F2 positive 30
F2 positive 67,90
Positive
Positive
F3 positives
QTY: 1
DOB: 07-03-2019
ID: 34
QTY: -
DOB: -
ID: -
F2 positive 6
F3 positive 67,10
Positive
Positive
Fn positives
QTY: 1
DOB: 07-06-2019
ID: 37
QTY: 2
DOB: 07-06-2019
ID: 40,41

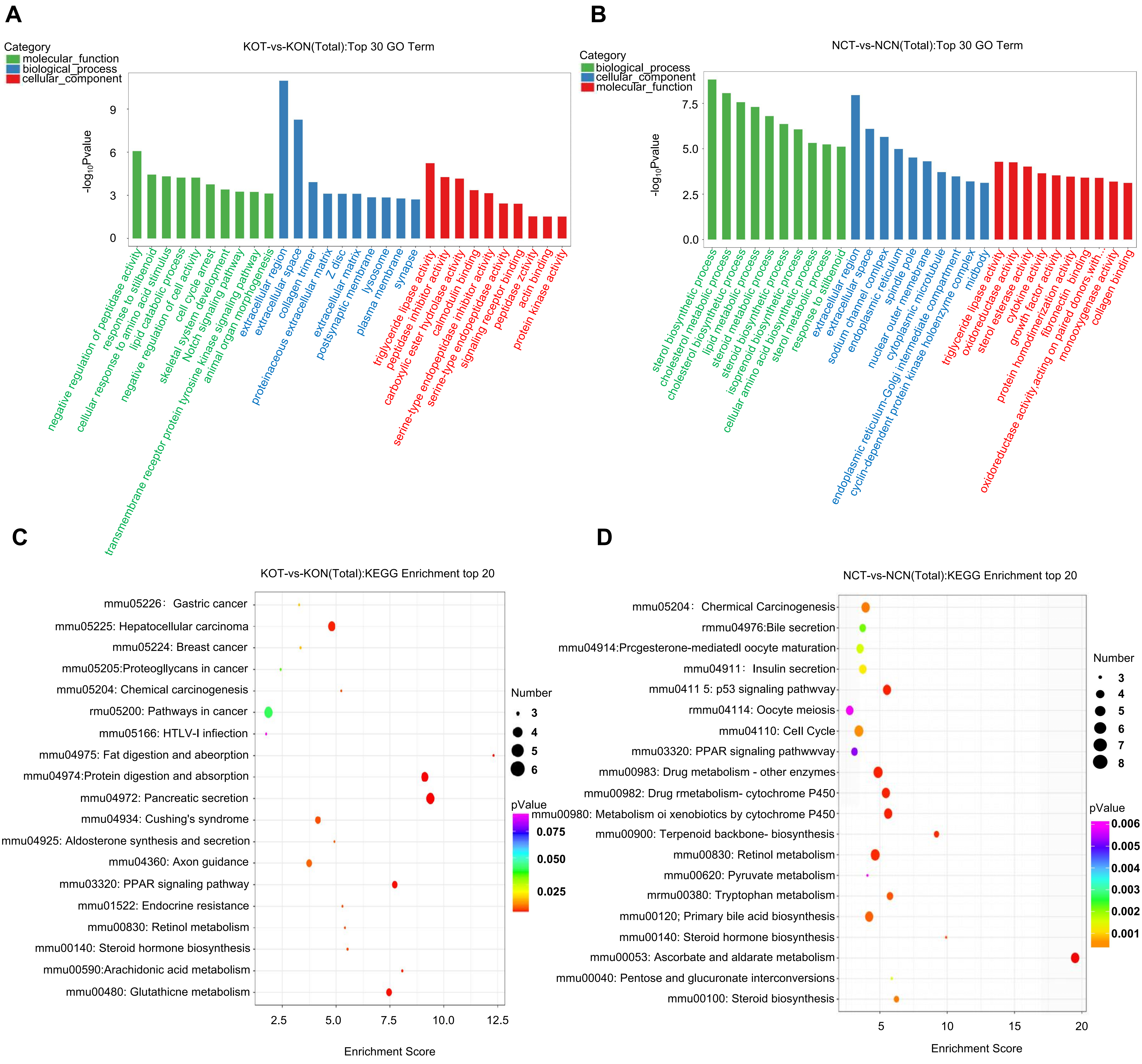
A
KOT-vs-KON(Total):Top 30 GO Term
Category
molecular_function
biological_process
cellular_component
-log10Pvalue
0
3
6
9
negative regulation of peptidase activity
response to stilbenoid
cellular response to amino acid stimulus
lipid catabolic process
negative regulation of cell activity
cell cycle arrest
skeletal system development
Notch signaling pathway
transmembrane receptor protein tyrosine kinase signaling pathway
animal organ morphogenesis
extracellular region
extracellular space
proteinaceous extracellular matrix
collagen trimer
Z disc
extracellular matrix
postsynaptic membrane
lysosome
plasma membrane
synapse
triglyceride lipase activity
peptidase inhibitor activity
carboxylic ester hydrolase activity
calmodulin binding
serine-type endopeptidase inhibitor activity
serine-type endopeptidase activity
signaling receptor binding
peptidase zctivity
actin binding
protein kinase activity
B
NCT-vs-NCN(Total):Top 30 GO Term
Category
biological_process
cellular_component
molecular_function
-log10Pvalue
0.0
2.5
5.0
7.5
sterol biosynthetic process
cholesterol metabolic process
cholesterol biosynthetuc process
lipid metabolic process
steroid metabolic process
steroid biosynthetic process
isoprenoid biosynthetic process
cellular amino acid biosynthetic process
sterol metabolic process
response to stilbenoid
extracellular region
extracellular space
sodium channel complex
endoplasmic reticulum
spindle pole
nuclear outer memebrane
cytoplasmic microtubule
endoplasmic reticulum-Golgi intermediate compartment
cyclin-dependent protein kinase holoenzyme complex
midbody
triglyceride lipase activity
oxidoreductase activity
sterol esterase activity
cytokine activity
growth factor activity
protein homodimerization activity
fibronectin binding
oxidoreductase activity,acting on paired donors,with...
monooxygenase activity
collagen binding
C
KOT-vs-KON(Total):KEGG Enrichment top 20
mmu05226：Gastric cancer
mmu05225: Hepatocellular carcinoma
mmu05224: Breast cancer
mmu05205:Proteogllycans in cancer
mmu05204: Chemical carcinogenesis
rmu05200: Pathways in cancer
mmu05166: HTLV-I infiection
mmu04975: Fat digestion and abeorption
mmu04974:Protein digestion and absorption
mmu04972: Pancreatic secretion
mmu04934: Cushing's syndrome
mmu04925: Aldosterone synthesis and secretion
mmu04360: Axon guidance
mmu03320: PPAR signaling pathway
mmu01522: Endocrine resistance
mmu00830: Retinol metabolism
mmu00140: Steroid hormone biosynthesis
mmu00590:Arachidonic acid metabolism
mmu00480: Glutathicne metabolism
2.5
5.0
7.5
10.0
12.5
Enrichment Score
Number
3
4
5
6
pValue
0.075
0.050
0.025
D
NCT-vs-NCN(Total):KEGG Enrichment top 20
mmu05204：Chemical Carcinogenesis
rmmu04976:Bile secretion
mmu04914:Prcgesterone-mediatedl oocyte maturation
mmu04911：Insulin secretion
mmu0411 5: p53 signaling pathwvay
rmmu04114: Oocyte meiosis
mmu04110: Cell Cycle
mmu03320: PPAR signaling pathwwvay
mmu00983: Drug metabolism - other enzymes
mmu00982: Drug rmetabolism- cytochrome P450
mmu00980: Metabolism oi xenobiotics by cytochrome P450
mmu00900: Terpenoid backbone- biosynthesis
mmu00830: Retinol metabolism
mmu00620: Pyruvate metabolism
mrmu00380: Tryptophan metabolism
mmu00120; Primary bile acid biosynthesis
mmu00140: Steroid hormone biosynthesis
mmu00053: Ascorbate and aldarate metabolism
mmu00040: Pentose and glucuronate interconversions
mmu00100: Steroid biosynthesis
5
10
15
20
Enrichment Score
Number
3
4
5
6
7
8
pValue
0.006
0.005
0.004
0.003
0.002
0.001

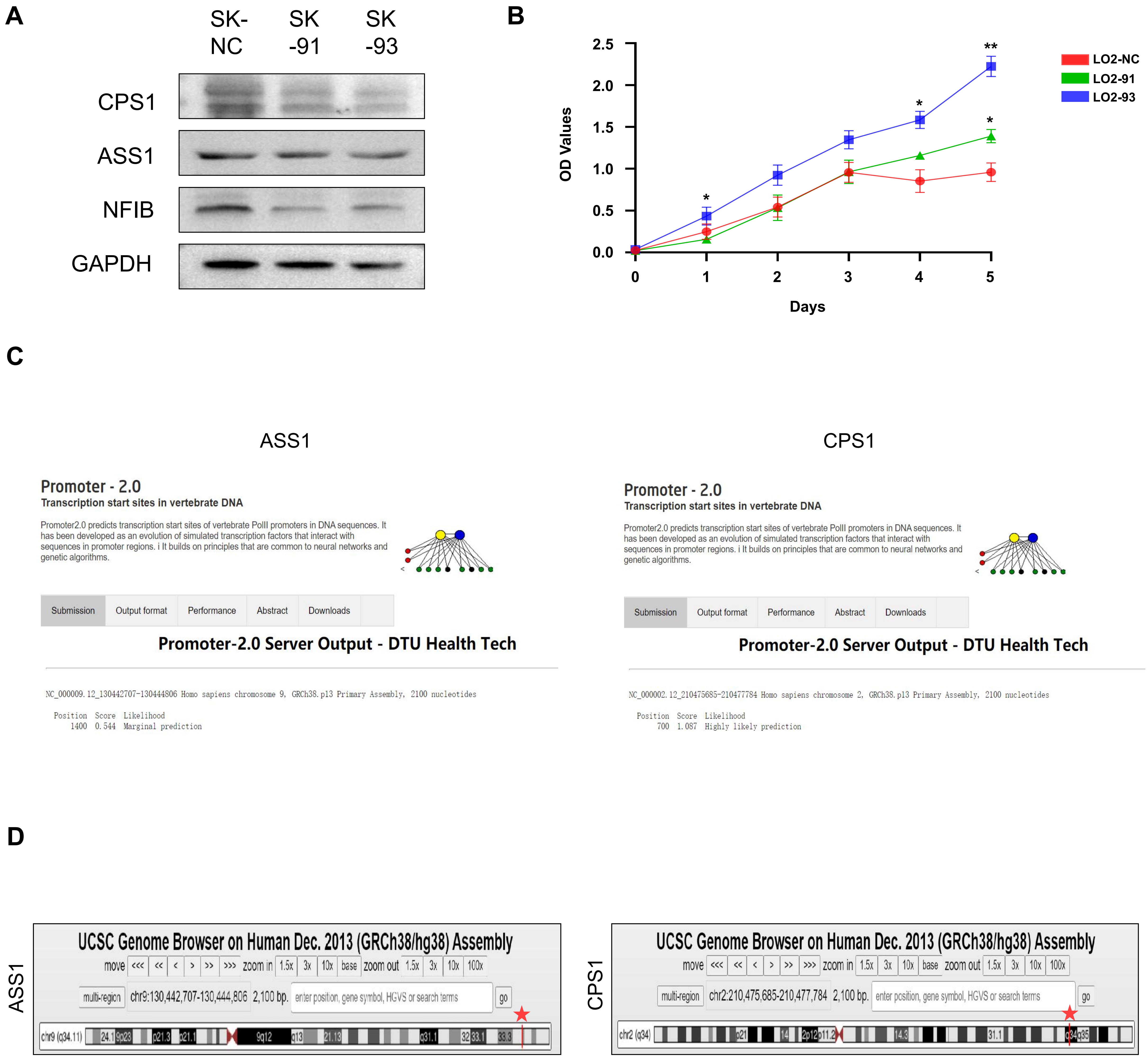

A
SK-NC
SK-91
SK-93
CPS1
ASS1
NFIB
GAPDH
B
OD Values
Days
LO2-NC
LO2-91
LO2-93
*
*
**
*
C
ASS1
Promoter - 2.0
Transcription start sites in vertebrate DNA
Promoter2.0 predicts transcription start sites of vertebrate PolII promoters in DNA sequences. It has been developed as an evolution of simulated transcription factors that interact with sequences in promoter regions. i It builds on principles that are common to neural networks and genetic algorithms.
Submission
Output format
Performance
Abstract
Downloads
Promoter-2.0 Server Output - DTU Health Tech
NC_000009.12_130442707-130444806 Homo sapiens chromosome 9, GRCh38.p13 Primary Assembly, 2100 nucleotides
Position Score Likelihood
1400 0.544 Marginal prediction
CPS1
Promoter - 2.0
Transcription start sites in vertebrate DNA
Promoter2.0 predicts transcription start sites of vertebrate PolII promoters in DNA sequences. It has been developed as an evolution of simulated transcription factors that interact with sequences in promoter regions. i It builds on principles that are common to neural networks and genetic algorithms.
Submission
Output format
Performance
Abstract
Downloads
Promoter-2.0 Server Output - DTU Health Tech
NC_000002.12_210475685-210477784 Homo sapiens chromosome 2, GRCh38.p13 Primary Assembly, 2100 nucleotides
Position Score Likelihood
700 1.087 Highly likely prediction
D
ASS1
UCSC Genome Browser on Human Dec. 2013 (GRCh38/hg38) Assembly
move <<< << < > >> >>> zoom in 1.5x 3x 10x base zoom out 1.5x 3x 10x 100x
multi-region chr9:130,442,707-130,444,806 2,100 bp. enter position, gene symbol, HGVS or search terms go
chr9 (q34.11)
CPS1
UCSC Genome Browser on Human Dec. 2013 (GRCh38/hg38) Assembly
move <<< << < > >> >>> zoom in 1.5x 3x 10x base zoom out 1.5x 3x 10x 100x
multi-region chr2:210,475,685-210,477,784 2,100 bp. enter position, gene symbol, HGVS or search terms go
chr2 (q34)

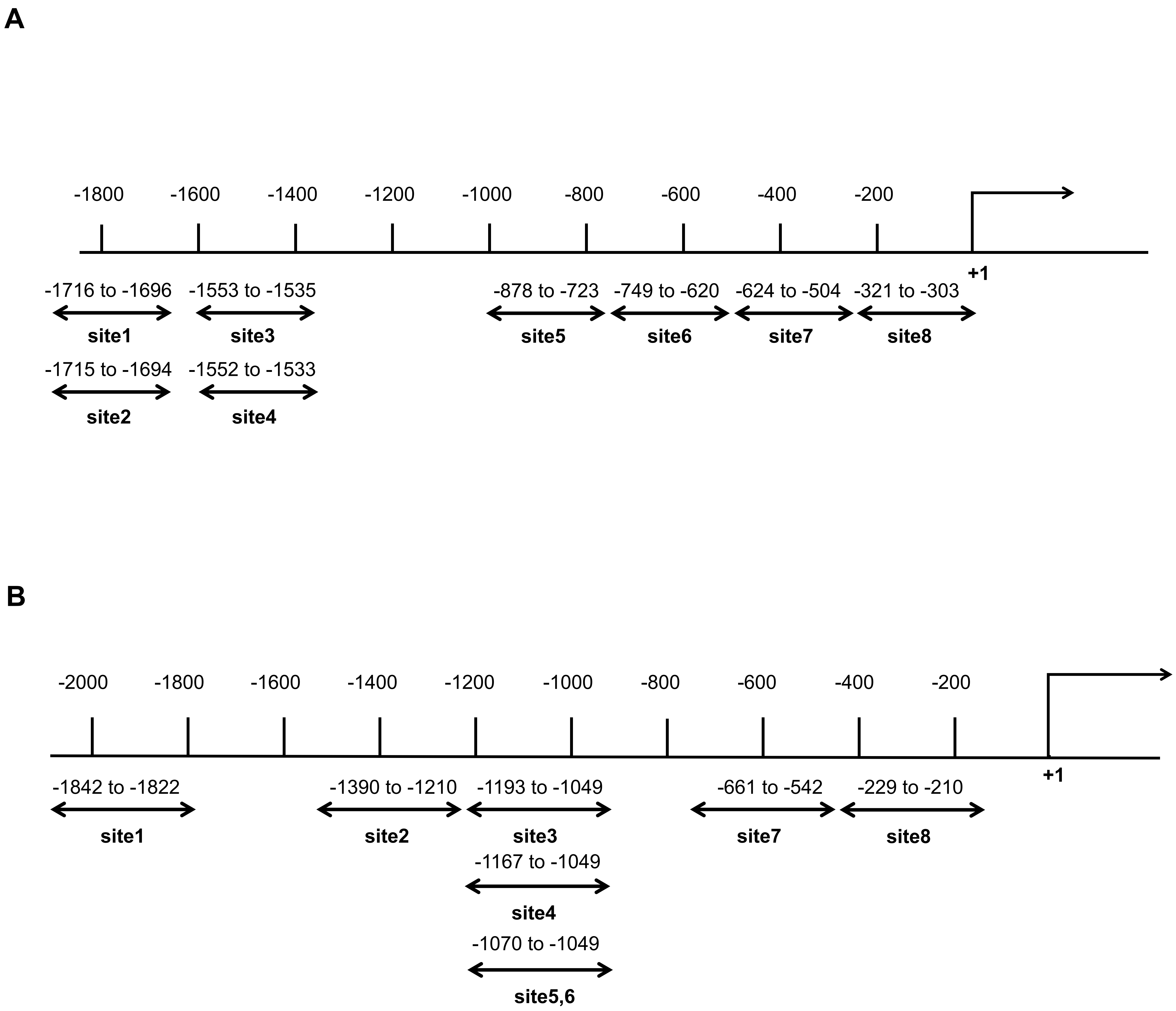

A
-1800
-1600
-1400
-1200
-1000
-800
-600
-400
-200
+1
-1716 to -1696
site1
-1553 to -1535
site3
-878 to -723
site5
-749 to -620
site6
-624 to -504
site7
-321 to -303
site8
-1715 to -1694
site2
-1552 to -1533
site4
B
-2000
-1800
-1600
-1400
-1200
-1000
-800
-600
-400
-200
+1
-1842 to -1822
site1
-1390 to -1210
site2
-1193 to -1049
site3
-661 to -542
site7
-229 to -210
site8
-1167 to -1049
site4
-1070 to -1049
site5,6
